# Spinal Meningitis

**Published:** 1883-07

**Authors:** 


### VII.—SPINAL MENINGITIS.

REPORTED FROM DR. BOWER'S INFIRMARY.

A gray horse, aet 12, had been sick for twelve days. Two or three days before this attack fairly set in the animal was seen to stagger in his gait. After that he was found in his stall one morning unable to rise.

He was at this time placed in a sling and raised to his feet, and kept there by its aid. All power of motion in the posterior extremities was suspended, accompanied by spasmodic contractions of the anterior extremities. After placing the animal in the slings, anodynes and anti-spasmodics were employed ; the bladder was emptied by means of a catheter. It was impossible to give a cathartic, either in a ball or drench. Catharses, however, was obtained by 15 ♏ of Oleum Tiglii, mixed with ʒii of Oleum-Olivæ. By drawing the tongue well forward the menstrum was placed well back upon the root; the indrawing of the tongue carried it well back, followed by a most satisfactory result.

When the acute inflammatory symptoms had subsided, the anodines and anti-spasmodics were discontinued. The horse was placed upon Nux Vomica and Chlorate of Potass, ʒi. of the former and ʒii. of the latter in powder, one each night, containing the above amount.

A favorable prognosis had been given.

The improvement was steady up to the 12th day, when he accidentally slipped from the sling. He was immediately replaced. This accident, however, proved a slight draw back in the previous steady progress toward recovery.

By the end of December, or about two weeks from the onset of the disease, the animal had completely recovered.

---

### VIII.—SIMPLE LAMENESS.

REPORTED FROM DR. ROBERTSON'S CLINIC HELD AT HIS INFIRMARY.

Aged bay horse, 15½ hands high.

One morning the animal was found in the stable and could not readily back out. This patient is in the habit of getting cast. The night before last he was apparently all right.

*Present Symptoms.*

There is swelling at the stiffle; a marked unwillingness to flex the joint; and pain upon pressure. When standing the leg is slightly flexed, with toe in advance of the opposite one.

The diagnosis is stiffle lameness, due to some sudden injury.

The prognosis in such cases is generally favorable.

The treatment which I should advise would be to first bathe the part with hot water, say twice daily, and then apply the following:

℞

| | |
|---|---|
| Aquæ Ammoniæ Fort., . . | ʒvi. |
| Olei Terabinthinæ, . . | ℥i. |
| Tr. Capsici, . . . . | ʒi. |
| Ether Sulphuric, . . . | ʒss. |
| Linamenti saponis, . . . | ℥viii. |

M sig. ex. use.

This liniment to be applied twice daily after the fomentations. Later, improvement was recorded.

